# The Role of Toll-Like Receptor 4 in Infectious and Noninfectious Inflammation

**DOI:** 10.1155/2016/6978936

**Published:** 2016-05-18

**Authors:** Monica Molteni, Sabrina Gemma, Carlo Rossetti

**Affiliations:** Dipartimento di Biotecnologie e Scienze della Vita, Università degli Studi dell'Insubria, Via J. H. Dunant 3, 21100 Varese, Italy

## Abstract

Toll-like receptor 4 (TLR4) belongs to the family of pattern recognition receptors (PRRs). They are highly conserved receptors that recognize conserved pathogen-associated molecular patterns (PAMPs), thus representing the first line of defense against infections. TLR4 has been long recognized as the sensing receptor for gram-negative lipopolysaccharide (LPS). In addition, it also binds endogenous molecules produced as a result of tissue injury. Hence, TLR4 represents a key receptor on which both infectious and noninfectious stimuli converge to induce a proinflammatory response. TLR4-mediated inflammation, triggered by exogenous or endogenous ligands, is also involved in several acute and chronic diseases, having a pivotal role as amplifier of the inflammatory response. This review focuses on the research progress about the role of TLR4 activation in infectious and noninfectious (e.g., sterile) inflammation and the effects of TLR4 signaling in some pathological conditions.

## 1. Introduction

The first function described for TLR4 was the recognition of exogenous molecules from pathogens (pathogen-associated molecular pattern molecules (PAMPs)), in particular the molecules from gram-negative bacteria (e.g., LPS) [1]. Recently, it has been widely demonstrated that TLR4 is also involved in the recognition of endogenous molecules released by injured tissues and necrotic cells. These molecules, called damage-associated molecular pattern molecules (DAMPs), induce the activation of a strong proinflammatory response through interaction with TLR4 [2]. Generally, inflammation has a protective role. It is a complex and coordinated process followed by the induction of resolution pathways that restore tissue integrity and function. However, in some cases, excessive and/or poorly regulated inflammatory response can be harmful for the organism. In several diseases with microbial (gram-negative infections) or nonmicrobial etiology (ischemia/reperfusion injury and neurodegenerative and neurological diseases) there is an involvement of TLR4 activation that, under certain circumstances, can contribute to disease progression.

## 2. TLR4 Signaling

**Figure 1:**
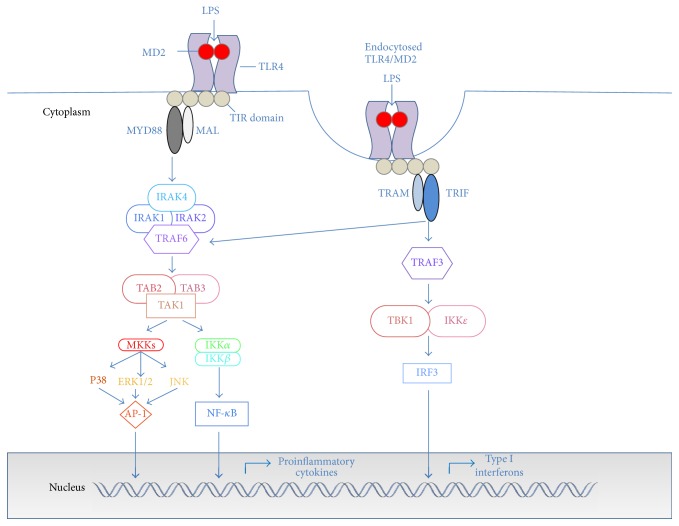
TLR4 intracellular signaling pathways. TLR signaling is triggered by ligand-induced dimerization of the receptors. TIR domains of TLR4 recruit TIR domain-containing adaptor proteins MyD88 and MAL (MyD88-dependent pathway) or TRIF and TRAM (MyD88-independent pathway). MyD88-dependent pathway involves the recruitment and the activation of IRAKs (IRAK1, IRAK2, and IRAK4) and TRAF6 that induce TAK1 activation. TAK1, in turn, leads to MAP kinase kinase- (MKK-) mediated activation of MAPKs (p38, JNK, and ERK1/2) and activation of IKK complex. MAPKs and IKK complex induce activation and translocation in the nucleus of transcription factors such as NF-*κ*B and AP-1. MyD88-independent pathway involves TRIF and TRAM adaptor proteins and, via TRAF3, the recruitment of TBK1/IKK*ε*, followed by the activation and translocation in the nucleus of the transcription factor IRF3. MyD88-dependent pathway induces production of proinflammatory cytokines, and MyD88-independent pathway induces the production of type I interferons.

TLR4 is expressed on the cell surface on both hematopoietic and nonhematopoietic cells, including endothelial cells [3], cardiac myocytes [4], and cells of the central nervous system (CNS) [5]. TLR4 is composed of a 608-residue extracellular domain and a 187-residue intracellular domain that is involved in the intracellular signaling cascade [6]. It has been demonstrated that transfection of TLR4 alone is not enough for LPS recognition, and physical association of TLR4 with myeloid differentiation 2 (MD2) on the cell surface is required for ligand-induced activation [7–9]. MD2 lacks transmembrane and intracellular domains and noncovalently associates with the extracellular domain of TLR4 by interaction with LPS, forming the TLR4/MD2 receptor complex [10]. Detailed crystallographic data are reported elsewhere [11–13]. Other accessory molecules that enhance LPS sensing are LPS-binding protein (LBP) and CD14 that favor the transferring of LPS monomers to MD2 and TLR4 [14]. After LPS binding, a dimerization of two TLR4/MD2 complexes occurs, resulting in conformational changes of the TLR4 homodimer that induce the recruitment of adaptor proteins containing Toll/interleukin-1 receptor-like (TIR) domains. These adaptors associate with the TLR4 cluster through homophilic interactions between TIR domains in the cytoplasmic tail of TLR4 and those present on the adaptors. Four adaptor proteins, involved in two distinct intracellular signaling pathways, have been described: myeloid differentiation primary response protein 88 (MyD88), MyD88-adaptor-like (MAL) protein, also known as TIR domain-containing adaptor protein (TIRAP), TIR domain-containing adaptor inducing IFN-*β* (TRIF), also known as TIR domain-containing adaptor molecule-1 (TICAM-1), and TRIF-related adaptor molecule (TRAM) [15]. MyD88-mediated signaling occurs mainly at the plasma membrane and involves a rapid recruitment of MyD88 and MAL proteins. Engagement of these adaptor molecules stimulates the recruitment and the activation by phosphorylation of IL-1R-associated kinases (IRAKs), the association of TNF-receptor-associated factor 6 (TRAF6), and the downstream activation of transforming growth factor *β*-activated kinase 1 (TAK1), mediated by the adaptor proteins, TAK1-binding protein 2 and TAK1-binding protein 3 (TAB2 and TAB3). TAK1 in turn activates the mitogen-activated protein kinases (MAPKs), JUN N-terminal kinase (JNK), p38, extracellular signal-regulated kinases (ERK1/2), and the IkB kinase complex (IKK), leading to the activation of important transcription factors, such as nuclear factor-*κ*B (NF-*κ*B) and activator protein-1 (AP-1), that promote the production of proinflammatory cytokines [15] (Figure 1). Activation of MyD88-independent pathway occurs in the endosomal compartment after internalization of the TLR4-MD2 complex. It involves the recruitment of adaptor proteins TRIF and TRAM, activation of TNF receptor-associated factor 3 (TRAF3), and the induction of IFN regulatory factor 3 (IRF3) nuclear translocation, mediated by tank-binding kinase 1 (TBK) and IKK*ε*. IRF3 transcription factor promotes the production of type I IFNs (Figure 1). CD14 favors receptor complex internalization [14], even though also CD14-independent translocation of the receptor complex to the endosome and TRIF signaling has been recently demonstrated [16].

Beyond the induction at the transcriptional level of proinflammatory mediators, TLR4 interaction with LPS also orchestrates the induction of mediators such as microRNAs (miRNAs) that posttranscriptionally regulate the shutdown of the proinflammatory response and induce a state of temporary refractoriness to further LPS stimulation. Therefore, a tight regulation of TLR4 signaling is important in tissue homeostasis to avoid excessive inflammation and to induce tissue repair following infection or injury [17, 18].

## 3. TLR4 Ligands

Since the discovery of the role of TLR4 as a sensor of bacterial LPS by Poltorak et al. [19] and Qureshi et al. [20], several other ligands have been identified from both exogenous sources from both the host tissues and cells (Table 1).

Exogenous ligands (PAMPs) are molecules isolated from bacteria, viruses, fungi, plants, and cyanobacteria. Most are agonists of TLR4/MD2 complex [19–28]. However, from bacteria and cyanobacteria (*Rhodobacter* and* Oscillatoria* species, resp.), TLR4 antagonists have also been obtained and their mechanism of action has been well characterized [29, 30]. These antagonists were employed both* in vitro* and* in vivo* in animal models of diseases, allowing investigation of the effects of TLR4 signaling modulation [29, 30].

Endogenous ligands (DAMPs) belong to two main groups: (a) molecules originated from extracellular matrix and [31–35] (b) intracellular mediators passively released or actively secreted by cells [36–49]. Even though for some DAMPs the ability to activate TLR4-mediated signaling has been questioned [50] and the mechanisms of interaction with TLR4/MD2 have not been investigated yet (no crystallographic data are available), there is no doubt that some endogenous molecules could use TLR4 to induce a proinflammatory response. A prototypic molecule of the extracellular matrix that can induce TLR4-mediated inflammation is the glycosaminoglycan hyaluronan. In normal conditions, hyaluronan is present in tissues in a high molecular form (up to 10^6^ Da). After tissue injury, it is degraded into small fragments, which have been shown to activate macrophages via TLR4 both* in vitro* and* in vivo* [31, 51]. Endogenous intracellular triggers of TLR4 include the DNA-binding protein high-mobility group box 1 (HMGB1) and cellular heat shock proteins (HSPs). After cell damage and necrosis, these molecules are released in the extracellular milieu, thus inducing a strong proinflammatory response mediated by TLR4 [36, 40, 52]. Beyond its role in sterile inflammation, HMGB1 is also actively released by immunocompetent cells after exposure to the products of pathogenic bacteria, thus representing a common mediator at the intersection of infectious and noninfectious inflammatory response [36]. Only for HMGB1, among endogenous TLR4 ligands, more detailed studies using surface plasmon resonance were done to confirm the specific binding of HMGB1 to TLR4 [53].

## 4. The Role of TLR4 in Infectious Diseases

The central role played by TLR4 in gram-negative infections comes from studies on TLR4-mutated or TLR4-deficient mice [54]. It has been observed that TLR4-mutated strain C3H/HeJ is hyporesponsive to LPS and highly susceptible to infection by gram-negative bacteria such as* Salmonella typhimurium* and* Neisseria meningitidis* [54–56]. On the other hand, studies demonstrated that TLR4^−/−^ mice were protected from endotoxin shock induced by* E. coli*, thus supporting TLR4 as a possible target for therapeutic intervention in sepsis [57]. In humans, genetic studies on TLR4 polymorphisms (missense mutations D299G and T399I) gave conflicting results. Some studies have linked TLR4 polymorphisms to an increased susceptibility to sepsis due to gram-negative infection; other studies failed to confirm this (reviewed in [58]). Furthermore, results with primary cells isolated from individuals according to D299G/T399I haplotypes did not show any difference in LPS responsiveness [59]. More recent studies have shown a single nucleotide polymorphism rs11536889 in 3′-untranslated region of TLR4 associating with periodontitis [60] and organ failure in sepsis [61]. This polymorphism was shown to affect expression of TLR4 on the cell surface and IL8 production in response to LPS possibly through miRNA regulation [62].

Given the role of TLR4 in the activation of the proinflammatory response during infection, pharmacological approaches targeting TLR4 have been developed with the aim to control host's deleterious proinflammatory response called “cytokine storm” occurring in the early phase of sepsis. Unfortunately, the results of clinical trials with molecules targeting TLR4 were disappointing [63], suggesting that immune suppression, which follows the “cytokine storm,” represents the leading process in the progression of sepsis [64].

## 5. The Role of TLR4 in Noninfectious Diseases

### 5.1. Ischemia/Reperfusion (I/R) Injury

Tissue I/R injury is caused by a sudden interruption of the blood supply to an organ followed by its restoration. Hypoxia induces cell injury and tissue damage with the release of several DAMPs, including HMGB1 [65]. Reperfusion is essential to preserve the organ; however, the exposure of the ischemic area to restored blood flow can lead to an acute inflammatory response causing an additional extensive tissue destruction, a phenomenon termed “reperfusion injury.” DAMPs released by necrotic and distressed cells, through interaction with PRRs, induce the release of proinflammatory mediators by resident macrophages and dendritic cells that recruit, in the reperfusion phase, neutrophils, monocyte, and lymphocytes from blood flow to the ischemic organ. Indeed, the recruitment and activation of neutrophils produce the release of reactive oxygen and nitrogen species and of proteolytic enzymes that are highly cytotoxic and exacerbate tissue damage [66]. Acute myocardial and cerebral infarctions, as well as solid organ transplantations, are all conditions in which I/R injury occurs. In myocardial infarction, reperfusion injury accounts for up to 50% of myocardial infarct size, thus reducing the beneficial effects of reperfusion [67]. In organ transplantation, I/R injury resulting from cold preservation and warm reperfusion of the transplanted graft directly contributes to allograft rejection [68]. Several studies in animal models clearly demonstrated that TLR4, being the target of several DAMPs, plays a key role in I/R injury [69–74]. A great deal of information about the role of TLR4 in I/R injury came from studies using TLR4-mutated (C3H/HeJ) or TLR4^−/−^ mice. In experimental models of acute myocardial infarction, TLR4 mutant mice showed a reduction of infarct size, decreased activation of NF-*κ*B and AP-1, and lower myocardial mRNA levels of interleukin-1*β* (IL-1*β*), monocyte chemotactic factor-1 (MCP-1), and interleukin-6 (IL-6), in comparison with wild-type mice [75]. In TLR4^−/−^ mice we observed reduced infarct sizes and polymorphonuclear cells infiltrating the ischemic area (unpublished observations). The importance of TLR4 was also highlighted by studies of cold I/R injury in syngeneic heart transplant between TLR4 mutant and wild-type mice. The results demonstrated that TLR4 signaling on both donor and recipient cells contributes to systemic and intragraft inflammatory response [76]. Interesting results have also been obtained using a synthetic inhibitor of TLR4/MD2 complex (Eritoran) and a natural cyanobacterial TLR4 antagonist in a murine model of myocardial I/R injury. The results suggest that the downregulation of TLR4-induced proinflammatory response has beneficial effects in reducing tissue damage [77, 78] and this was associated, in the experiments with the cyanobacterial antagonist, with a reduced number of polymorphonuclear leukocytes infiltrating the ischemic area [78]. Confirming results about the positive effects of TLR4 targeting in I/R injury were also obtained in experimental models of hepatic I/R injury in which decreased inflammatory mediators and inhibition of HMGB1 release from hepatocytes were observed after treatment with Eritoran [79]. Indeed, no data are available about the impact of TLR4 signaling inhibition in I/R injury on the long-term tissue repair. Our preliminary data (unpublished results) in a mouse model of acute myocardial infarction using the cyanobacterial TLR4 antagonist suggest that early inhibition of TLR4 signaling just before reperfusion positively affects tissue remodeling, since long-term cardiac function was better in mice treated with the antagonist in comparison to mice treated with vehicle. However, further in-depth studies are needed to clarify the role of TLR4 signaling in tissue repair.

### 5.2. Neurodegenerative and Neurological Diseases

Neuroinflammation is the common hallmark of several neurodegenerative and neurological diseases [80–82]. In the CNS, microglial cells are resident phagocytes that constantly control the extracellular environment and sense for the presence of pathogens or injured cells. Microglial cells are the immunological “sentinels” of the CNS: they express TLR4 and are highly responsive to LPS* in vitro* [83]. Microglial activation by noxious stimuli represents a defensive response with the aim to restore tissue homeostasis. In several pathological conditions, however, persistent exposure to danger signals can cause aberrant microglia activation with the production of proinflammatory mediators and the release of reactive oxygen and nitrogen species that result in neuronal dysfunction and/or neuronal cell loss. It has been reported that direct TLR4 stimulation with LPS produces immediate and long-term memory deficits in mice models of endotoxemia, especially in aged mice [84, 85]. In similar experiments, the contribution of HMGB1 on memory impairment mediated by both TLR4 and receptor for advanced glycation end products (RAGE) has been demonstrated [85, 86]. Increased expression of TLR4 in microglial cells has been observed in animal models and patients of Alzheimer's disease (AD) [87–89], Parkinson's disease (PD) [90], and Amyotrophic Lateral Sclerosis (ALS) [91, 92].

In AD, amyloid-*β* (A*β*) oligomers directly induce microglial activation through several receptors, including TLR4 [49, 80]. Activated microglia has an active role not only in the production of proinflammatory mediators but also in the phagocytosis of A*β*. Indeed, the continuous formation of A*β* caused, at least in part, by positive feedback between inflammation and amyloid precursor protein processing drives a chronic detrimental and nonresolving proinflammatory loop. In this contest, the role of TLR4 is not clear, probably due to the complex mechanisms controlling reacting microglia phenotypes [80, 93].* In vitro* studies showed a role of TLR4 in the A*β*-induced neurotoxicity [87, 88]. Differently, some experimental studies using transgenic AD mice carrying mutated TLR4 showed reduced microglial activation but also reduced A*β* clearance with exacerbation of cognitive deficits [94]. These data suggest that there is a delicate balance between the production of proinflammatory mediators and A*β* phagocytosis in glial cells, and an inefficient clearance of A*β*, only partially TLR4-dependent, could be involved in disease progression [80, 93, 95, 96]. In support of this hypothesis, Michaud et al. [97] recently demonstrated that treatment of transgenic AD mice with monophosphoryl lipid A (MPLA), a LPS-derived analog of gram-negative lipid A, led to a significant reduction of A*β* accumulation in the brain and enhanced cognitive function. MPLA is a TLR4 agonist but does not induce large amounts of proinflammatory mediators. In this context, MPLA was shown to induce a potent phagocytic response by microglia while triggering a moderate inflammatory response* in vivo*.

In PD, where accumulation of extracellular misfolded *α*-synuclein is observed, contrasting results about the role of TLR4 have been obtained. In a mouse model of PD induced by 1-methyl-4-phenyl-1,2,3,6-tetrahydropyridine (MPTP), TLR4 was shown to mediate cell death of dopaminergic neurons and TLR4-deficient mice were partially protected against MPTP toxicity [98]. Indeed, experimental TLR4 deficiency was shown to decrease *α*-synuclein clearance* in vitro* by murine microglia [99, 100].

The studies about the role of TLR4 in other neurodegenerative and neurological diseases, ALS and epilepsy, gave more clear results. In ALS upregulation of TLR4 and cytoplasmic HMGB1 were observed in reactive glia (astrocytes and microglia) and neurons of the spinal cord in ALS patients [91]. In a mouse genetic model of ALS (superoxide dismutase 1-mutant mice), chronic administration of LPS (once every 2 weeks for 3 months) in presymptomatic mice accelerated motor neuron degeneration and disease progression [101]. Furthermore, De Paola et al. [92] demonstrated that the chronic treatment with a TLR4 antagonist in a mouse model of spontaneous motor neuron degeneration (wobbler mice) produced potent anti-inflammatory effects (reduction of gliosis and TNF-*α* production) with significant improvements of motor functional tests. In epilepsy, analyses of hippocampal specimens obtained at surgery from patients with drug-resistant temporal lobe epilepsy showed increased TLR4 and HMGB1 expression in glial cells (astrocytes) and neurons [102]. Moreover, in acute and chronic mice models of seizures a proconvulsant pathway involving TLR4-HMGB1 axis was demonstrated. Intriguingly, antagonists targeting TLR4 were shown to delay seizure onset and decrease acute and chronic seizure recurrence [102].

## 6. Conclusions

Since its discovery, a great deal of experimental data supported TLR4 as a key player of the inflammatory process due to both infectious and noninfectious stimuli. In several pathological conditions TLR4 engagement contributes to disease resolution; however, when TLR4 activation pathways are poorly regulated, it can contribute to disease progression. More information is needed to clarify the role of TLR4 engagement in the different phases of I/R injury or during the neurodegenerative processes, with a particular attention to the effects of TLR4 signaling on the fine phenotypic changes occurring* in vivo* in both peripheral (macrophages) and CNS (microglia) innate immune cells. In this framework, TLR4 targeting could represent a successful means to manipulate macrophages and glial cells activation and the development of molecules acting on TLR4 could represent new disease-modifying therapeutic agents for the treatment of I/R injury or for neurodegenerative diseases.

## Figures and Tables

**Table 1:** TLR4 ligands.

TLR4 ligands	Activity	References
*Exogenous natural ligands*	****	
Lipopolysaccharides from gram-negative bacteria	Agonist	[20]
F protein of syncytial virus	Agonist	[21]
Mannuronic acid polymers from gram-negative bacteria	Agonist	[22]
Teichuronic acid from gram-positive bacteria	Agonist	[23]
*Chlamydia pneumoniae* HSP60	Agonist	[24]
Flavolipin from* Flavobacterium meningosepticum*	Agonist	[25]
Mannan from *S. cerevisiae* and *C. albicans*	Agonist	[26]
*Dengue virus* NS1 protein	Agonist	[27]
Plant paclitaxel	Agonist	[28]
Lipopolysaccharides from *Rhodobacter *sp.	Antagonist	[29]
Lipopolysaccharide-like (CyP) from *Oscillatoria *sp.	Antagonist	[30]

*Endogenous ligands*		
*Extracellular matrix ligands*		
Hyaluronan	Agonist	[31]
Biglycan	Agonist	[32]
Fibronectin	Agonist	[33]
Heparan sulphate	Agonist	[34]
Tenascin-C	Agonist	[35]
*Intracellular and secreted endogenous ligands*	****	
HMGB1	Agonist	[36]
HSP22	Agonist	[37]
HSP60	Agonist	[38]
HSP70	Agonist	[39]
HSP72	Agonist	[40]
HSP70L1	Agonist	[41]
HSP Gp96	Agonist	[42]
Calcineurin B	Agonist	[43]
*β*-defensin 2	Agonist	[44]
S100 proteins	Agonist	[45]
Surfactant protein A	Agonist	[46]
Resistin	Agonist	[47]
Fibrinogen	Agonist	[48]
Amyloid-*β*	Agonist	[49]
